# Detection of Herpesviruses in Wild Bird Casualties in Slovenia

**DOI:** 10.3389/fvets.2022.822212

**Published:** 2022-02-25

**Authors:** Zoran Žlabravec, Brigita Slavec, Al Vrezec, Urška Kuhar, Olga Zorman Rojs, Zlatko Golob, Jožko Račnik

**Affiliations:** ^1^Institute of Poultry, Birds, Small Mammals, and Reptiles, Faculty of Veterinary Medicine, University of Ljubljana, Ljubljana, Slovenia; ^2^Department for Organism and Ecosystems Research, National Institute of Biology, Ljubljana, Slovenia; ^3^Slovenian Museum of Natural History, Ljubljana, Slovenia; ^4^Institute of Microbiology and Parasitology, Faculty of Veterinary Medicine, University of Ljubljana, Ljubljana, Slovenia; ^5^Golob d.o.o. Clinic for Small, Wild, and Exotic Animals, Shelter for Protected Wildlife, Muta, Slovenia

**Keywords:** wildlife, herpesvirus, avian, polymerase chain reaction, Slovenia

## Abstract

The complete host range of avian herpesviruses in wild birds is unknown, and information about nucleotide sequences is available only in limited cases. The aim of this study was to detect the presence of herpesviruses in wild birds and to gain more information about their phylogenetic relationship. Oropharyngeal and cloacal swabs from 447 wild birds from 15 different orders presented as wildlife casualties were examined for herpesvirus presence with PCR targeting a fragment of the DNA polymerase gene. Herpesviruses were detected in oropharyngeal and/or cloacal swabs in 34 (7.5%) birds belonging to 11 species from six different avian orders: Accipitriformes, Charadriiformes, Columbiformes, Falconiformes, Passeriformes, and Strigiformes. The results of phylogenetic analysis showed that various herpesviruses sequences are present in the wild bird population. Some herpesviruses are host species–specific, whereas in some cases very similar sequences were detected through different avian orders, which confirms findings that herpesviruses are not always restricted to bird species. It seems that herpesvirus transmission could occur by predation from avian prey, and even by superpredation—for example, large owls, such as the Eurasian eagle owl (*Bubo bubo*) or Ural owl (*Strix uralensis*), preying on smaller raptors. This can lead to greater infection exposure and is in line with the fact that raptors were the most infected species group. Nevertheless, the individual or simultaneous detection of herpesviruses in oropharyngeal and cloacal swabs shows that both swab samples should be used for herpesvirus detection in wild birds.

## Introduction

Avian herpesviruses cause some of the more familiar diseases of birds, such as Marek disease (1, 2), infectious laryngotracheitis (3), duck plague in poultry (4), and Pacheco disease in parrots (5), as well as diseases in free-living birds that are equally important. However, because of several disadvantages regarding disease surveillance in free-living birds, including obtaining biological samples, virus infections in these animals are less studied. Generally, many herpesviruses replicate in healthy birds with little or no apparent signs of infection, but under certain environmental conditions various forms of disease associated with high rates of mortality may occur. Clinical signs of herpesvirus infections in wild birds may comprise a broad spectrum of non-specific signs ranging from respiratory to enteric problems, such as depression, reduced/absent appetite, regurgitation, biliverdinuria, diarrhea, conjunctivitis, or sudden death (6).

Pigeons are the natural hosts of pigeon herpesvirus 1, in the new nomenclature also known as columbid herpesvirus 1 (CoHV-1) (7). In infected pigeon flocks, mature birds are asymptomatic carriers and some of them may intermittently shed virus (8). CoHV-1 causes the disease known as Smadel's disease or ingluvinitis of pigeons, a contagious disease of predominantly young pigeons of racing and fancy breeds or immunocompromised adult pigeons (6). Numerous field studies and detection of CoHV-1 in raptors suggest that consumption of infected prey species, in particular pigeons, is most likely the source of herpesvirus infections in hawks, eagles, and owls (9–11). Furthermore, CoHV-1 was also detected in other non-raptorial birds (12), and recent studies have shown that many additional herpesviruses, which differ from CoHV-1, have been identified in various live-free living birds such as owls and songbirds (13–15). In birds of prey, the disease caused by CoHV-1 is known as herpesvirus hepatitis or inclusion body disease, and in some cases it is described as fatal, with mortality approaching 100% (11). Disease outbreaks and also mortality due to herpesvirus infections have been reported among free-ranging aquatic birds, marine birds, and waterfowl (16–19). Fatal cases of animals infected with passerid herpesviruses have also been reported in songbirds from aviary enclosures in America and Canada (20, 21). In general, severe and fatal disease caused by herpesvirus infections in birds are mostly described in interspecies infections, whereas in intraspecies infections clinical signs are mild and followed by a period of latency (22).

Based on biological properties and genomic attributes, the Herpesviridae have been divided into three subfamilies: Alphaherpesvirinae, Betaherpesvirinae, and Gammaherpesvirinae (23). The Alphaherpesvirinae subfamily is divided into five genera; namely, *Iltovirus, Mardivirus, Scutavirus, Simplexvirus*, and *Varicellovirus* (24). For universal detection of unknown herpesviruses in birds and mammals, a PCR with pan-herpes degenerate primers for detection of the highly conserved herpesvirus DNA polymerase gene showed good results in previous studies (10, 12–14, 25). All avian herpesvirus analyzed to date are phylogenetically most closely related to the members of the Alphaherpesvirinae subfamily; however, it should be emphasized that, although herpesvirus infections have been described in various species of free-living birds, only limited herpesvirus nucleotide (nt) sequence data are available in free-living birds.

This study further extends insight into the spread of herpesviruses in wild avian hosts from various orders, studies their phylogenetic relationship, and presents more information about the ubiquitous features of herpesviruses in the free-living bird population.

## Materials and Methods

### Bird Species and Samples

Cloacal and oropharyngeal swabs were collected from 447 wild birds treated at the Clinic for Birds, Small Mammals, and Reptiles, Faculty of Veterinary Medicine, University of Ljubljana as wildlife casualties for veterinary diagnosis, treatment, and care between October 2017 and December 2019 (Table 1). A clinical examination was performed by a veterinarian after admission. Samples were collected from live avian patients during the clinical procedure or were taken while birds were anesthetized using inhalational isoflurane and oxygen delivered via mask to facilitate clinical examination and diagnostics. All efforts were made to minimize animal stress and discomfort. Sterile dry swabs (Copan, Italy) were used to separately swab the oropharynx and cloaca of live birds. Swabs were stored in a refrigerator at 4°C up to 48 h until analyzed.

**Table 1:** Detection of herpesvirus by avian order, family, and species.

**Order**	**Family**	**Genus and species**	**Common name**	**% HV positive**	***n***
Accipitriformes *n* = 44	Accipitridae *n* = 44	*Aquila chrysaetos*	Golden eagle	100	1
		*Buteo buteo*	Common buzzard	16	32
		*Accipiter gentilis*	Northern goshawk	0	1
		*Accipiter nisus*	Eurasian sparrowhawk	0	7
		*Circaetus gallicus*	Short-toed snake eagle	0	1
		*Pernis apivorus*	European honey buzzard	0	2
Anseriformes *n* = 7	Anatidae *n* = 7	*Anas platyrhynchos*	Mallard	0	2
		*Cygnus olor*	Mute swan	0	5
Apodiformes *n* = 2	Apodidae *n* = 2	*Apus apus*	Common swift	0	2
Charadriiformes *n* = 6	Laridae *n* = 2	*Larus michahellis*	Yellow-legged gull	50	2
	Scolopacidae *n* = 4	*Scolopax rusticola*	Eurasian woodcock	0	4
Ciconiiformes *n* = 1	Ciconiidae *n* = 1	*Ciconia ciconia*	White stork	0	1
Columbiformes *n* = 109	Columbidae *n* = 109	*Columba livia domestica*	Domestic pigeon	18	89
		*Columba palumbus*	Common wood pigeon	20	5
		*Streptopelia decaocto*	Eurasian collared dove	0	1
		*Streptopelia turtur*	European turtle dove	0	14
Bucerotiformes *n* = 1	Upupidae *n* = 1	*Upupa epops*	Eurasian hoopoe	0	1
Falconiformes *n* = 34	Falconidae *n* = 34	*Falco peregrinus*	Peregrine falcon	0	1
		*Falco subbuteo*	Eurasian hobby	0	1
		*Falco tinnunculus*	Common kestrel	3	32
Galliformes *n* = 1	Phasianidae *n* = 1	*Phasianus colchicus*	Common pheasant	0	1
Gruiformes *n* = 3	Rallidae *n* = 3	*Fulica atra*	Eurasian coot	0	1
		*Rallus aquaticus*	Water rail	0	2
Passeriformes *n* = 183	Corvidae *n* = 52	*Corvus corax*	Common raven	0	2
		*Corvus cornix*	Hooded crow	7	40
		*Coloeus monedula*	Western jackdaw	0	3
		*Garrulus glandarius*	Eurasian jay	0	2
		*Pica pica*	Eurasian magpie	0	5
	Emberizidae *n* = 1	*Emberiza calandra*	Corn bunting	0	1
	Fringillidae *n* = 17	*Carduelis carduelis*	European goldfinch	0	6
		*Spinus spinus*	Eurasian siskin	0	1
		*Chloris chloris*	European greenfinch	33	3
		*Coccothraustes coccothraustes*	Hawfinch	0	5
		*Fringilla coelebs*	Common chaffinch	0	2
	Hirundinidae *n* = 7	*Delichon urbicum*	Common house martin	0	1
		*Hirundo rustica*	Barn swallow	0	6
	Motacillidae *n* = 1	*Motacilla alba*	White wagtail	0	1
	Muscicapidae *n* = 6	*Erithacus rubecula*	European robin	0	5
		*Muscicapa striata*	Spotted flycatcher	0	1
	Paridae *n* = 10	*Periparus ater*	Coal tit	0	1
		*Parus major*	Great tit	0	8
		*Poecile palustris*	Marsh tit	0	1
	Passeridae *n* = 33	*Passer domesticus*	House sparrow	0	33
	Sittidae *n* = 2	*Sitta europaea*	Eurasian nuthatch	0	2
	Sturnidae *n* = 8	*Sturnus vulgaris*	Common starling	0	8
	Sylviidae *n* = 1	*Sylvia atricapilla*	Eurasian blackcap	0	1
	Turdidae *n* = 45	*Turdus merula*	Common blackbird	0	40
		*Turdus philomelos*	Song thrush	0	5
Pelecaniformes *n* = 5	Ardeidae *n* = 5	*Ardea cinerea*	Grey heron	0	4
		*Botaurus stellaris*	Eurasian bittern	0	1
Piciformes *n* = 7	Picidae *n* = 7	*Dendrocopos major*	Great spotted woodpecker	0	3
		*Drycopus martius*	Black woodpecker	0	1
		*Picus canus*	Grey-headed woodpecker	0	2
		*Picus viridis*	European green woodpecker	0	1
Podicipediformes *n* = 2	Podicipedidae *n* = 2	*Podiceps cristatus*	Great crested grebe	0	1
		*Podiceps nigricollis*	Black-necked grebe	0	1
Strigiformes *n* = 42	Strigidae *n* = 42	*Asio otus*	Long-eared owl	43	7
		*Athene noctua*	Little owl	0	2
		*Bubo bubo*	Eurasian eagle owl	25	4
		*Otus scops*	Eurasian scops owl	0	4
		*Strix aluco*	Tawny owl	0	17
		*Strix uralensis*	Ural owl	12	8
Total				34	447

The free-living birds belonged to 15 different orders; bird taxonomy follows Gill et al. (26): Accipitriformes (*n* = 44 individuals), Anseriformes (*n* = 7), Apodiformes (*n* = 2), Bucerotiformes (*n* = 1), Charadriiformes (*n* = 11), Ciconiiformes (*n* = 1), Columbiformes (*n* = 109), Falconiformes (*n* = 34), Galliformes (*n* = 1), Gruiformes (*n* = 3), Passeriformes (*n* = 183), Pelecaniformes (*n* = 5), Piciformes (*n* = 7), Podicipediformes (*n* = 2), and Strigiformes (*n* = 42). More detailed information is shown in Table 1.

### DNA Extraction and PCR

Cloacal and oropharyngeal swabs were individually vortexed in 2 ml phosphate-buffered saline for 1 min and supernatant was stored for nucleic acid extraction. In each assay, a DNA of a alphaherpesvirus-positive sample was included as a positive control. As negative control extracted phosphate-buffered saline was used.

Total DNA and RNA were extracted from 140 μl of samples using the QIAamp Viral RNA Mini Kit (Qiagen, Germany) according to the manufacturer's instructions. PCR was used to detect diverse herpesvirus as previously described by VanDevanter et al. (27). The degenerated primers DFA (5′-GAYTTYGCNAGYYTNTAYCC-3′), ILK (5′-TCCTGGACAAGCAGCARNYSGCNMTNAA-3′) and KG1 (5′-GTCTTGCTCACCAGNTCNACNCCYTT-3′) targeted the DNA polymerase protein. This procedure was followed by a nested-PCR with the primers TGV (5′-TGTAACTCGGTGTAYGGNTTYACNGGNGT-3′) and IYG (5′-CACAGAGTCCGTRTCNCCRTADAT-3′). The PCR volume was 20 μl, and it contained 10 μl of 2 × DreamTag Green PCR Master Mix (Thermo Scientific, Europe), 1 μM of each PCR primer, 2 μl of isolated DNA, and deionized water up to 20 μl. The parameters for primer and nested PCR were denaturation at 95°C for 5 min, followed by 45 cycles of denaturation at 94°C for 30 s, annealing at 46°C for 1 min, extension at 72°C for 1 min, and final extension at 70°C for 7 min.

### Detection and Sequencing of PCR Products

Amplified products were separated by electrophoresis on 1.8% agarose gel (Sigma-Aldrich, USA) containing ethidium bromide. PCR products of 215 to 315 bp were excised and purified with a FastGene Gel/PCR extraction kit (Nippon Genetics, Europe) and sent for sequencing to the Macrogen Laboratory (Macrogen Inc., Netherlands).

### Phylogenetic Analysis

The nucleotide sequences obtained were first analyzed by BLAST (28) to identify sequences relevant for further analyses within the NCBI database. Nucleotide alignments were constructed in Geneious Prime 2019 v1.3 software suite (Biomatters Ltd., Auckland, New Zealand) with MAFFT translation alignment (29). Phylogenetic analysis was performed using the maximum likelihood method with the Tamura 3-parameter model and 1,000 bootstrap replicates by MEGA 7.0 (30). The genetic distances among sequences were calculated using the *p*-distance model (pairwise distance) in MEGA 7.0.

## Results

### PCR and Sequencing

The presence of herpesviruses in clinical samples was investigated by PCR. A PCR product was detected in swabs of 34 out of 447 live free-living birds. In four birds, herpesvirus was detected in oropharyngeal and cloacal swabs, in three birds herpesvirus was detected only in cloacal swabs, and in 27 birds herpesvirus was detected only in oropharyngeal swabs. Herpesvirus was found in 11 species from six different avian orders: Accipitriformes, Charadriiformes, Columbiformes, Falconiformes, Passeriformes, and Strigiformes (Tables 1, 2).

**Table 2:** Details of HV sequences obtained in this study.

**Host species**	**ID number**	**Sample tested positive**	**Sequence acc. number**	**Sequence length (bp)**
Common buzzard (*Buteo buteo*)	241/17	Oropharynx	MW533123	231
Common buzzard (*Buteo buteo*)	245/17	Oropharynx	MW533124	235
Common buzzard (*Buteo buteo*)	298/18	Oropharynx	MW533125	216
Common buzzard (*Buteo buteo*)	1062/18	Oropharynx, cloaca	MW533126	222
Common buzzard (*Buteo buteo*)	594/19	Oropharynx	MW533127	228
Golden eagle (*Aquila chrysaetos*)	367/19	Oropharynx	MW533128	228
Common kestrel (*Falco tinnunculus*)	338/18	Cloaca	MW533129	210
Long-eared owl (*Asio otus*)	690/19	Oropharynx	MW533132	231
Long-eared owl (*Asio otus*)	750/19	Oropharynx	MW533133	228
Long-eared owl (*Asio otus*)	969/19	Oropharynx	MW533134	228
Eurasian eagle owl (*Bubo bubo*)	353/19	Oropharynx	MW533135	210
Ural owl (*Strix uralensis*)	1544/19	Oropharynx	MW533137	228
Yellow-legged gull (*Larus michahellis*)	831/19	Oropharynx	MW533138	219
Hooded crow (*Corvus cornix)*	416/18	Oropharynx	MW533139	231
Hooded crow (*Corvus cornix)*	417/18	Oropharynx, cloaca	MW533140	201
Hooded crow (*Corvus cornix)*	115/19	Cloaca	MW533141	228
European greenfinch (*Chloris chloris*)	19/19	Oropharynx	MW533143	204
Domestic pigeon (*Columba livia domestica*)	238/17	Oropharynx	MW625922	225
Domestic pigeon (*Columba livia domestica*)	31/18	Oropharynx	MW625923	234
Domestic pigeon (*Columba livia domestica*)	229/18	Oropharynx	MW625925	225
Domestic pigeon (*Columba livia domestica*)	293/18	Oropharynx	MW625926	234
Domestic pigeon (*Columba livia domestica*)	294/18	Oropharynx, cloaca	MW625927	225
Domestic pigeon (*Columba livia domestica*)	314/18	Oropharynx	MW625928	231
Domestic pigeon (*Columba livia domestica*)	392/18	Oropharynx	MW625929	225
Domestic pigeon (*Columba livia domestica*)	1665/18	Oropharynx	MW625930	234
Domestic pigeon (*Columba livia domestica*)	11/19	Oropharynx	MW625931	219
Domestic pigeon (*Columba livia domestica*)	136/19	Oropharynx	MW625934	207
Domestic pigeon (*Columba livia domestica*)	183/19	Oropharynx	MW625935	234
Domestic pigeon (*Columba livia domestica*)	231/19	Oropharynx	MW625936	225
Domestic pigeon (*Columba livia domestica*)	255/19	Cloaca	MW625937	225
Domestic pigeon (*Columba livia domestica*)	633/19	Oropharynx	MW625938	225
Domestic pigeon (*Columba livia domestica*)	903/19	Oropharynx, cloaca	MW625939	234
Domestic pigeon (*Columba livia domestica*)	904/19	Oropharynx	MW625940	210
Common wood pigeon (*Columba palumbus*)	360/19	Oropharynx	MW626272	222

### Phylogenetic Analysis

**Figure 1:**
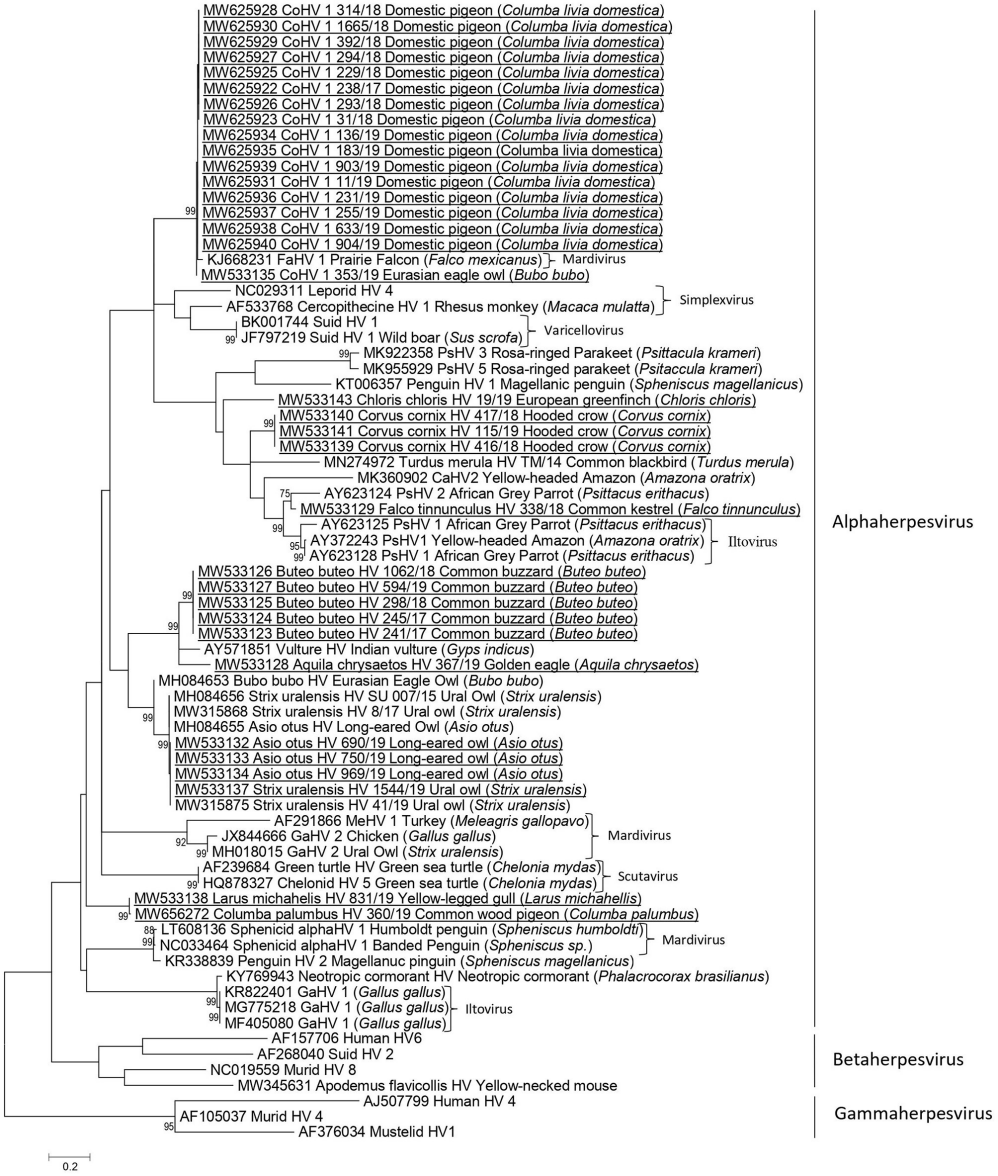
Phylogenetic relationship based on partial DNA polymerase gene nucleotide sequences of herpesviruses from wild birds in Slovenia and other herpesviruses derived from the GenBank database. The scale bar indicates substitutions per site. Nucleotide sequences obtained in the current study are additionally underlined.

Nucleotide sequences of the partial DNA polymerase gene of herpesviruses were obtained from 34 herpesvirus-positive samples and used for phylogenetic analysis. Phylogenetic comparison of herpesvirus nt sequences from Slovenian free-living birds from this study and other avian and mammal alpha-, beta-, and gammaherpesvirus revealed high phylogenetic diversity among alphaherpesvirus (Table 2, Figure 1). All herpesvirus nt sequences investigated in this study clustered with alphaherpesviruses, with nt identity ranging from 49.8 to 100% among them.

Herpesvirus sequences detected in domestic pigeons (*Columba livia domestica*) (238/17, 31/18, 229/18, 293/18, 294/18, 314/18, 392/18, 1,665/18, 11/19, 136/19, 183/19, 231/19, 255/19, 633/19, 903/19, 904/19) shared 99.51 to 100% nt identity and clustered together with CoHV-1 detected in an Eurasian eagle owl (*Bubo bubo*) (353/19), and with FaHV-1 detected in a prairie falcon (*Falco mexicanus*) with 97.53 to 99.02% and 97.55 to 98.04% nt identity, respectively.

The herpesvirus sequence detected in a common wood pigeon (*Columba palumbus*) (360/19) was distinct from the herpesvirus sequences detected in domestic pigeons, with 60.78 to 61.27% nt identity and clustered together with a herpesvirus sequence detected in a yellow-legged gull (*Larus michahellis*) (831/19), with 99.51% identity between them.

In owls, the herpesvirus nucleotide sequences belonged to two genetically distinct groups sharing 56.8% nt identity. The herpesvirus sequence (353/19) detected in a Eurasian eagle owl (*Bubo bubo)* was very similar to the CoHV-1 group of sequences in domestic pigeons in this study (97.53 to 99.02% identity), whereas the herpesviruses detected in a Ural owl (*Strix uralensis*) (1,544/19) and in long-eared owls (*Asio otus*) (690/19, 750/19/, 969/19) were identical and grouped together with the herpesviruses detected in a Eurasian eagle owl (MH084653) and in long-eared owls (MH084655), sharing from 90.74 to 100% nt identity.

In common buzzards (*Buteo buteo*), identical herpesvirus sequences (594/19, 245/17, 241/17, 298/18, 1,062/18) were detected and were most similar to herpesviruses detected in an Indian vulture (*Gyps indicus*), with 83.33% identity, and golden eagle (*Aquila chrysaetos*), with 75.98% identity.

Among falcons, the herpesvirus nt sequence detected in a common kestrel (*Falco tinnunculus*) (338/18) clustered together with the PsHV-1/PsHV-2 group with 81.59 to 86.07% nt identity and was most closely related to PSHV-2 (AY623124) detected in an African gray parrot (*Psittacus erithacus*), with 86.07% identity.

In songbirds, herpesvirus nt sequences detected in hooded crows (*Corvus cornix*) (416/18, 115/19, 417/19) were 100% identical and shared 76.12% nt identity with the closest relative; namely, the herpesvirus nt sequence of the common kestrel (338/18) detected in our study. The nt sequence of herpesvirus detected in a European greenfinch (*Chloris chloris*) (19/19) shared 68.16% nt identity with nt sequences of herpesviruses detected in hooded crows in this study (416/18, 115/19, 417/19).

## Discussion

This study describes the detection of novel and known herpesviruses in free-living avian species that are most similar to bird alphaherpesviruses according to a phylogenetic analysis based on partial DNA polymerase sequences. The occurrence of herpesviruses in free-living bird species in our study was 7.6%, with 34 out of 447 birds testing positive. Generally, different studies have shown different levels of occurrence of herpesvirus in free-living bird species. In owls, herpesvirus was detected at levels from 9.41% (16/170) (15) to 14.5% (8/55) (13), and in seabirds from 3.8% (4/104) (31) to 5.6% (14/250) (32), whereas a lower presence of herpesvirus was detected in passerine birds, at only 0.8% of positive samples (4/525) (14). In studies that examined free-living birds from different orders, the highest occurrence of herpesvirus (20.4%; 18/88) was seen in a study from Poland, where the presence of herpesvirus was detected in domestic pigeons, birds of prey, and non-raptorial birds (12). A lower prevalence was detected in a study from Australia, where 403 birds from 13 different genera were investigated and herpesvirus was detected in only three out of 409 birds (0.7%) (33). The differences in herpesvirus occurrence detected among different bird species may arise from varying study approaches. The major reasons could be the detection of herpesvirus in dead birds, which was performed in most studies (12, 13, 31, 33), and the use of different PCR assay tests. However, other reasons should also be considered, such as the biology and ecology of the bird species tested, intermittent shedding of herpesviruses via the cloaca and/or trachea, and the immune status of infected birds (34).

Although sampling of birds presented to a veterinary clinic as wildlife casualties is a useful method for studying viruses in free-living birds, it should be considered that the inherently biased population of birds (e.g., injured birds, particular locations, and common species in an area) (33) presented to a veterinary facility could have impact on virus detection. Herpesviruses are known for their subclinical or latent infections, in which under certain conditions—particularly conditions which we may have encountered, such as collision/trauma and/or concurrent infections—can trigger reactivation, causing recurrent infection or asymptomatic shedding and consequently detection of herpesvirus in clinical samples (swabs). However, detection of herpesvirus in oropharyngeal and/or cloacal swabs in this study could indicate the detection of herpesvirus in an initial or reactivated phase of infection.

In raptorial birds, herpesvirus DNA was detected in 12 out of 121 birds (Accipitriformes 13.6%, 6/44; Falconiformes 2.9%, 1/34; and Strigiformes 11.9%, 5/42). Herpesviruses were detected in golden eagle (1/1), common buzzards (5/32), common kestrel (1/32), Eurasian eagle owl (1/4), long-eared owls (3/7), and Ural owl (1/8). The prevalence in susceptible species ranged from 16 to 100%.

Herpesvirus is the etiological agent responsible for inclusion body disease or herpesvirus hepatitis in hawks, falcons, and owls (9, 10). The disease is fatal, with mortality approaching to 100% (35). In the eagle and falcon population, individual cases of herpesvirus infection have been reported in different species (12, 36–39); however, only limited sequence data are available. Previous reports showed that herpesvirus sequences detected in eagles, owls, and falcons are very similar if not identical to the pigeon herpesvirus (CoHV-1) and that the consumption of infected pigeons or infected birds of prey is the most likely mode of transmission in raptors. The detection of CoHV-1 in a Eurasian eagle owl with 98.53 to 99.02% nt identity to CoHV-1 detected in domestic pigeons in this study could support the theory of prey-related herpesvirus transmission because the diet of the Eurasian eagle owl also includes potentially infected birds, especially in temperate regions and at low elevations (40). In Slovenia, for example, this may include known herpesvirus-infected bird species among birds of prey, pigeons, and corvids (41). However, detection and phylogenetic analysis of other herpesvirus sequences detected in three long-eared owls and one Ural owl in this study showed that these herpesvirus sequences have a distant relationship with known CoHV-1 and other alphaherpesvirus partial DNA polymerase sequences. These findings confirm the presence of different herpesvirus in owls (13), in which virus transmission between conspecifics was found to be more likely than transmissions from small mammals consumed as prey (15). However, interspecific disease transmission can take place at least occasionally due to intraguild superpredation; for example, when the Ural owl preys upon the long-eared owl (42). Herpesvirus sequences different from previously published ones were obtained also in falcons, eagles, and other bird orders.

In general, very different herpesvirus nt sequences of the partial DNA polymerase gene were detected among orders of raptors in this study. In our study, 51.7 to 54.7%, 60.29 to 63.3%, and 55.2 to 56.2% nt identities were shown between Acciptriformes and Falconiformes, Acciptriformes and Strigiformes, and Falconiformes and Strigiformes, respectively, and only one out of 12 herpesvirus nt sequences detected in raptors was similar to CoHV-1. The common kestrel herpesvirus DNA polymerase sequence was clustered together with known PsHV1 and PsHV2 detected in the African gray parrot and yellow-headed amazon, and it was different from previously detected CoHV-1 in various species of falcons, including the common kestrel (10, 12, 43). In raptors we have found clear herpesvirus clade divergence by grouping Accipitriformes and Strigifomes in one, and Falconiformes and Psittaciformes together with Passeriformes in other clade. This distinction is in line with avian phylogenetic relationships with Falconiformes being more related to Psittaciformes and Passeriformes than to other raptor groups (44). Apparently, herpesvirus evolution followed evolution history of their hosts rather than ecological convergences. Within Accipitriformes, herpesvirus sequences detected in five common buzzards were identical and were clustered together with the herpesvirus sequence detected in a golden eagle in our study and with the vulture herpesvirus detected in an Indian vulture (*Gyps indicus*). Even though raptors, Accipitriformes and Strigiformes, were the most infected group of species, even in this group we found herpesvirus-resistant species despite the higher number of individuals being examined—for example, sparrowhawks (*Accipiter nisus*) and especially tawny owls (*Strix aluco*)—which also agrees with previous studies (13, 15, 45).

It seems that herpesviruses in raptors are more or less species- or order-specific, and that they differ from herpesviruses detected in pigeons, which provide opposite results from previous reports, in which the hypothesis was that identical herpesviruses are detected in raptor and pigeon populations (10, 12, 33). Furthermore, they differ from herpesviruses detected in songbirds, showing 49.7 to 76.1% nt identity to herpesviruses detected in songbirds in this and previous reports (14), which excludes the possibility of songbirds as the source of herpesvirus infection in birds of prey and owls. There appear to be only two published reports on the detection and partial characterization of herpesvirus in wild passerine birds. In Poland, the viruses that were studied and detected in brain samples of the hooded crow and song thrush (*Turdus philomelos*) were classified as CoHV-1 through analysis of partial DNA polymerase gene sequences (12). A recent study of 525 herpesvirus-tested free-living passerine birds showed the presence of herpesvirus in two Eurasian blackcaps (*Sylvia atricapilla*), a common blackbird (*Turdus merula*), and a Eurasian blue tit (*Cyanistes caeruleus*) with relatively low nt identity between host species; however, within the same species (e.g., Eurasian blackcaps), identical herpesvirus sequences were detected (14). Similar results, with the detection of identical herpesvirus in three hooded crows and a diverse sequence detected in a European greenfinch, obtained in this study, could implicate the species-specific feature of herpesviruses in songbirds as well. Furthermore, both studies showed that herpesviruses different from CoHV-1 are present in passerine birds. The natural host range of avian herpesviruses is highly restricted, and most herpesviruses are thought to have evolved in association with a single host species (23). Although previous studies have detected different partial DNA polymerase sequences in owls and their prey (14, 15), some studies describe the detection of identical partial DNA polymerase sequences in different bird species, also suggesting the possible transmission of herpesvirus between species; for example, in domestic pigeons and raptors (10–13). Some studies also show that some species of large gulls could predate on other bird species, including feral pigeons (46–48). A very similar DNA polymerase sequence detected in a yellow-legged gull and common wood pigeon, where the difference in sequence identity could arise from an adaptation step in the emergence of host-switching viruses (49), could suggest that pigeons could be the source of herpesvirus infection; however, based on the dietary habits of the yellow-legged gull (50), this seems very unlikely. The results clearly show that the yellow-legged gull is susceptible to herpesviruses that differ from previously described CoHV-1 detected in brain samples in a dead herring gull (*Larus argentatus*) (12). Interestingly, the DNA polymerase sequence detected in a wood pigeon showed only 60.78 to 61.30% nt identity to CoHV-1 detected in the order Columbiformes in this study. All the CoHV-1 strains detected in domestic pigeons are genetically very similar (99.51 to 100%) to each other and cluster together with the CoHV-1 detected in a Eurasian eagle owl in this study and the CoHV-1 detected in a prairie falcon. These results align with the fact that domestic pigeons are considered a natural reservoir of CoHV-1 and are a potential source of infection for any susceptible native species that might coexist with them or consume them (51). However, in tawny owls no CoHV-1 was detected in this or previous studies (13, 15) even though they occasionally prey on domestic pigeons (52); this may be related to their polymorphism (13) or coevolved lower susceptibility to herpesvirus, but this requires further study.

In this study, the herpesvirus DNA polymerase sequence was readily detected in oropharyngeal and/or cloacal swabs. Although the herpesvirus presence in oropharyngeal swabs was detected with a higher detection rate than in cloacal swabs (81.6 vs. 18.4%), one cannot not rule out the importance of cloacal swabs in the detection of herpesvirus in wild birds, especially because in three out of 34 live wild birds herpesvirus was detected only in a cloacal swab. This could point to herpesvirus replicating poorly in the intestinal tract and/or kidneys, or to the herpesvirus detected in the cloaca shedding in the oropharyngeal region and then passing to and being digested through the digestive system. Nevertheless, these results suggest that wild birds with an unknown infection status and unknown duration of infection should be tested with a combination of oropharyngeal and cloacal swabs, which would maximize the probability of herpesvirus detection. However, even with this combination of sample types, it must be emphasized that the likelihood of herpesvirus-infected birds testing negative in both samples remains a possibility because the latent non-productive stage of herpesvirus in live wild birds is difficult to detect due to challenging (in some cases inaccessible) sampling of possibly latency sites of alphaherpesvirus, such as sensory ganglia or mononuclear cells (34, 53). Furthermore, the various herpesvirus sequences detected in this study that differ from known avian herpesvirus could mean that different tissue tropism and replication sites could also be present, as previously described for herpesvirus. This remains to be investigated.

This study has contributed valuable information regarding herpesvirus present in wild birds in Slovenia, and it has shown that very different herpesvirus sequences are present in the wild bird population, at least for the region of the DNA polymerase gene analyzed. Some detected herpesviruses could be prey-related, whereas others show a tendency to be order- or species-specific. In the future, full-length genome characterization should be performed to establish whether the (previously unknown) partial viral sequences are novel herpesviruses and to gain more information regarding the diversity of herpesvirus circulating in the wild bird population.

## Data Availability Statement

The datasets presented in this study can be found in online repositories. The names of the repository/repositories and accession number(s) can be found in the article/supplementary material.

## Ethics Statement

Ethical review and approval was not required for the animal study because all the diagnostic methods were made to provide proper treatment and care of injured (clinical) birds/patients. All the birds were treated at the Clinic for Birds, Small Mammals, and Reptiles, Faculty of Veterinary Medicine, University of Ljubljana as wildlife casualties.

## Author Contributions

ZŽ, AV, and JR: conceptualization and writing—original draft preparation. ZŽ, AV, BS, UK, ZG, and JR: methodology. ZŽ, AV, UK, and BS: software. BS: validation. ZŽ, AV, JR, BS, and OZR: investigation. ZŽ, AV, JR, BS, ZG, and OZR: resources. ZŽ, BS, AV, UK, JR, OZR, and ZG: writing—review and editing. All authors have read and agreed to the published version of the manuscript.

## Funding

This study was supported by the Slovenian Research Agency, Junior Researchers grant 50525, Environment and Food Safety project group P4-0092.

## Conflict of Interest

The authors declare that the research was conducted in the absence of any commercial or financial relationships that could be construed as a potential conflict of interest.

## Publisher's Note

All claims expressed in this article are solely those of the authors and do not necessarily represent those of their affiliated organizations, or those of the publisher, the editors and the reviewers. Any product that may be evaluated in this article, or claim that may be made by its manufacturer, is not guaranteed or endorsed by the publisher.
